# Drinking water behavior and willingness to use filters by middle-aged and elderly residents in rural areas: A cross-sectional study in Tengchong, China

**DOI:** 10.3389/fpubh.2022.961870

**Published:** 2022-09-20

**Authors:** Yuxin Duan, Ruiheng Wu, Haoqiang Ji, Xu Chen, Jia Xu, Yunting Chen, Meng Sun, Yuanping Pan, Ling Zhou

**Affiliations:** ^1^School of Public Health, Dalian Medical University, Dalian, China; ^2^School of Public Health, Shandong University, Jinan, China

**Keywords:** drinking water behavior, willingness to use filters, rural areas, middle-aged and elderly, unboiled tap water

## Abstract

Access to safe drinking water is critical to health and development issues, and residents' drinking behavior reflects their awareness of health and water hygiene. Random sampling and face-to-face questionnaires were used to investigate the drinking water behavior, sanitation and perceptions of drinking water among middle-aged and elderly residents in Tengchong, southwest Yunnan from July 1 to July 28, 2021. Differences between groups were assessed using the Chi-square test and *t*-test. Two binary logistic regression analyses were conducted to explore the influencing factors of drinking unboiled tap water and willingness to use filters. Results show that 35% of residents drink unboiled tap water, and 29.8% of respondents indicated a willingness to use filters. The model results showed a strong correlation between 60 and 79 years old (OR: 0.510, 95% CI: 0.303–0.858), 80 and above years old (OR: 0.118, 95% CI: 0.038–0.365), drinking water at a regular interval (OR: 0.397, 95% CI: 0.257–0.612), wanting to gain knowledge about drinking water (OR: 0.198, 95% CI: 0.099–0.395), Perceived health risks (PHR) (OR: 0.847, 95% CI: 0.771–0.929), having kidney stones (OR: 2.975, 95% CI: 1.708–5.253) and drinking unboiled tap water (*p* < 0.05). 60–79 years old (OR: 0.446, 95% CI: 0.244–0.815), 80 and above years old (OR: 0.228, 95% CI: 0.064–0.812), water storage (OR: 0.088, 95% CI: 0.026–0.300), middle school and above (OR: 2.238, 95% CI: 1.289–3.883), household water treatment (HWT) (OR: 33.704, 95% CI: 9.726–116.791), Perceived health risks (PHR) (OR:1.106, 95% CI: 1.009–1.213), water authority satisfaction (WAT) (OR:0.857, 95% CI: 0.769–0.956) and willingness to use filters were correlated (*p* < 0.05). Our findings suggested that a certain proportion of permanent middle-aged and elderly residents in rural areas still drink unboiled tap water, and residents are less willing to use filters. Residents' perception of drinking water can reflect residents' drinking water behavior and willingness to a certain extent. It is recommended that the government and Centers for Disease Control (CDC) should strengthen relevant measures such as knowledge popularization and health education, and regulate the water use behavior of middle-aged and elderly residents. Promote safe, economical and effective household water filtration facilities to ensure public health safety.

## Introduction

Access to safe drinking water is crucial to health and development issues. It is estimated that about a quarter of people in the home lack safely managed services of drinking water, and nearly half of the world's population lacks safety management of health facilities (1, 2). More than 200 million people in rural areas in China do not have access to safe drinking water due to limited water services and lack of complete water treatment facilities, compared to almost all cities with well-equipped water treatment facilities (3, 4). In addition, the accumulation of human and animal waste in rural areas and the abuse of chemical fertilizers and pesticides have exacerbated the problem of drinking water safety in rural areas (5, 6).

Due to China's geographical and economic constraints, many rural areas receive water through small centralized water supply systems (SCWS) (4). This type of water supply is provided and managed by the villages themselves, with few regular monitoring reports and a lack of proper water purification and disinfection facilities (7, 8). Yunnan Province, where the study area is located, has one of the lowest qualified rates of water quality in China (7). In 2013, a survey of 1,067 rural drinking water safety projects in Yunnan province showed that only 14.34% had complete treatment facilities, and only 66.83% of treated water samples were qualified (7). It is hard to find reliable and affordable safe water in rural areas. As a result, the burden of providing safe water falls on households. Tengchong, is dotted with hot springs and geothermal fields whose discharge can contaminate groundwater and surface water, putting residents' health at risk (9, 10).

In developed countries, tap water is subject to strict quality control and management (11, 12). In most cases, tap water can be directly drunk (12, 13). However, in China, due to poor water quality and secondary pollution, direct drinking tap water has certain risks (14). The habit of drinking boiled water, which greatly reduces the risk of diarrhea and other gastrointestinal diseases in China (15). However, there are still many rural residents with poor health habits and low health awareness, who drink unboiled tap water or even raw water. A survey conducted in Tengchong County found that the infection rate of *Entamoeba histolytica* among hospitalized patients was twice the average for the Chinese population, more than half were rural residents, and was highly associated with drinking unboiled tap water (16). Point-of-use household water treatment (HWT) is often considered a stop-gap solution, yet in many low-income rural areas, where households are still remain responsible for treating drinking water (2). Based on the evaluation criteria used, ceramic, bio-sand, and domestic water filters are considered to be the most effective and have the greatest potential for widespread and sustainable use in improving household water quality to reduce water-borne diseases and deaths (17).

Previous studies in developed countries have extensively investigated the influencing factors of residents' drinking water behavior and drinking water choice, including the use of bottled water, tap water, and filters (13, 17, 18). However, there are few studies on residential water use behavior in China (15, 19). In fact, access to safe drinking water and healthy water use behavior has become the primary demand and goal of the Chinese public and government. With the deepening of the aging degree, the outflow of the young rural labor force, there are a large number of rural left-behind middle-aged and elderly residents (20, 21), and they are low income, poor living conditions, inadequate social security, education level is generally not high, is a vulnerable group that cannot be ignored. To ensure drinking water quality and guide residents to drink correctly, it is vital to explore the influencing factors of drinking water behavior and choices for drinking water providers and the government.

On this basis, this research randomly selected the elderly residents of six townships, in Tengchong, Yunnan province as the research object to carry out a cross-sectional survey. Possible influencing factors such as gender, age, ethnicity, income, education, access to drinking water, and sanitation were investigated. We innovatively included residents' perceptions of drinking water in our study to provide a more comprehensive analysis. This study investigated the current situation of drinking behavior and filter use intention of middle-aged and elderly residents under the SCWS and further explored the influencing factors.

## Materials and methods

### Study design and setting

This cross-sectional face-to-face survey of respondents in six townships (Tengyue, Qingshui, Beihai, Zhonghe, Hehua, and Mangbang) in Tengchong, Yunnan province was conducted between July 1, 2021, and July 28, 2021. The sample size was calculated to achieve 90% power using an α of 0.05. We calculated the sample size according to the formula (22): n=Zα22π(1−π)δ2. n is the sample size, Zα22 is the abscissa of the normal curve that cuts off an area at the tails (1-α equals the desired confidence level, 95%), π is the ratio of residents drinking unboiled water, which was 45% based on the results of a pre-survey conducted in the study area in January 2021, and δ is the desired level of precision; we set it at 0.05. Thus, the sample size is calculated as *n* = $\frac{1.96^{2}\times 0.45\left(1-0.45\right)}{0.05^{2}}$ ≈380.3. At least 380 samples should be taken to tell if a difference in the interested parameter exists. Taking into account of potential non-response rate, which we desired <10%, making the sample size was 418 at least. To improve the stability of the study results as much as possible, we finally distributed a total of 538 questionnaires. There should be adequate power at all waves. The sampling process is based on the per capita GDP of each township in Tengchong. First, all the 18 townships of Tengchong were stratified into three economic levels: high, medium, and low, and two townships were then randomly selected from each of the levels. Finally, six different townships were selected from all the townships of Tengchong. Residents over the age of 45 who have lived locally for more than 1 year and used domestic water from SCWS were randomly selected. The great majority of adults under the age of 45 in rural areas were excluded from the survey as they have worked away from home for the long term, and have strong mobility so that they change their drinking habits and sources of drinking water. Besides, our research team members recruited college students who were familiar with the local dialect and had a medical background as interviewers. We trained the interviewers before conducting the formal face-to-face interview, and they were responsible for filling in the questionnaire. The respondents signed informed consent before each questionnaire survey.

### Questionnaire development

The questionnaire was designed based on a literature review and expert consultation. A pilot study of 10% sample size at the survey site was then carried out to verify its feasibility and to confirm the representative of influential factors in January 2021. Subsequently, the ambiguous questions were revised and the order of the questions was adjusted and deleted unreasonable questions were. The mainly collected data was (1) demographic characteristics such as gender, age, nationality, income, education, and monthly household income; (2) household water conditions and sanitary conditions, which included tap water source, whether to drink bottled water, the toilet type, whether the respondents had kidney stones, whether to treat water at the household level, whether to drink tea, water storage, the way of drinking water, whether want to gain knowledge about drinking water, whether to drink unboiled tap water and willingness to use filters; (3) perception of their home tap water, which included Environmental Concern (EC) (23), Area Satisfaction (AS) (24), Perceived Health Risks (PHR) (25), Perceived Water Quality (PWQ) (24, 25), Organoleptic Perceptions (OP) (25), and Water Authority Trust (WAT) (26). The above six scales used in the perception of tap water, which has been widely used and verified in previous studies in the same field and all of them show good reliability and validity in the different cultural backgrounds (Table 1). The scales have acceptable reliability and validity, and Cronbach's α were all above 0.90 in the current study. Each scale consists of corresponding items, and a five-point Likert scale was used to measure levels of agreement: (1) strongly disagree, (2) disagree, (3) neutrality, (4) agree, and (5) strongly agree. The items of each scale range from strongly disagree to strongly agree. The value of strongly disagree is 1, and the value of strongly agree is 5. The total score of the scale is summed up by item scores, and the higher the score is, the stronger the perception degree is.

**Table 1:** Perception of household tap water scales.

**Variables**	**Items**	**Description**
EC: Environmental Concern	EC1	If things continue on their present course, we will soon experience a major ecological catastrophe
	EC2	The problems of the environment are not as bad as most people think
	EC3	We are quickly using up the world's natural resources
	EC4	People worry too much about human progress harming the environment
	EC5	We are spending too little money on improving and protecting the environment
AS: Area Satisfaction	AS1	In general, I would be happy living in this area for the next 15 years
	AS2	If I had the opportunity, I'd rather live in another area
PHR: Perceived Health Risks	PHR1	There are health risks associated with drinking tap water in my home
	PHR2	I don't believe there is any possibility of becoming ill from drinking water straight from the tap
	PHR3	There are so many chemicals and additives in my home tap water that it must be unhealthy
	PHR4	My tap water will not harm anybody
PWQ: Perceived Water Quality	PWQ1	I don't believe the quality of my home tap water is that bad that it needs improvement
	PWQ2	My tap water is usually of high quality
OP: Organoleptic Perceptions	OP1	I am happy with the taste of my tap water
	OP2	I am happy with the color of my tap water
	OP3	I am happy with the odor of my tap water
WAT: Water Authority Trust	WAT1	I trust the Water Authority to manage any risk that may be associated with tap water use in our village
	WAT2	I trust the Water Authority to ensure water safety and quality
	WAT3	The Water Authority provides information that can be trusted

Variables were measured using 5-point Likert scales: (1) strongly disagree, (5) strongly agree.

### Statistical analysis

Questionnaires with non-rural residents, logical errors, or large amounts of missing data were removed and finally included in the study sample 500, and we used the software EpiData3.1 (EpiData Association, Odense, Denmark) using the double-entry method to set up a database to enter the questionnaire data. Quantitative data were described by means and standard deviations (SD). Categorical data were described by frequency and percentage. Chi-square tests were used to evaluate differences in the categorical data between different groups. All variables included in the models were tested multicollinearity, and they all have a variance inflation factor (VIF) of <10. However, initial testing of the logistic regression model that included the PWQ scale resulted in a poorly fitting model and suppression effects on other variables, and the VIF of PWQ is very close to 10 (VIF = 9.93, 1/VIF = 0.1007). This scale was removed for these analyses, and it was also removed from a previous study (18). We introduced variables with *p*-value < 0.05 in the single factor test into the logistic regression model. Binary logistic regressions were performed to determine the predictors of drinking unboiled tap water behavior of middle-aged and elderly residents and the willingness to use filters; Odds ratios (ORs) with 95% confidence intervals (CIs) were calculated. The level of statistical significance was *p*-value < 0.05. All statistical analyses were performed using Stata/MP version 16.0 (StataCorp, College Station, TX, USA).

## Results

### Demographic characteristics

Of the respondents, 35.00% of respondents drank unboiled tap water in daily life, and 29.80% showed a willingness to use filters. The average age of the respondents was 66.14 years old (SD = 9.19), and more than half of them (57.00%) were female. Most are between 60 and 70 years old, accounting for 39.80%. There are only 7.40% ethnic minorities, and the rest are all Han. Only 6.80% of residents have a monthly household income (MHI) of < 1,000 CNY, and 9.20% >10,000 CNY. The education level of the residents is generally low, a majority of respondents (72.00%) had a primary school and less education, and 28.00% had a middle school and above education. The chi-square test indicated that age and educational status were statistically significant with drinking unboiled tap water (*p* < 0.05), and age, income, and educational status were statistically significant with a willingness to use filters (p < 0.05) (Table 2).

**Table 2:** Drinking water behavior and willingness to use filters by demographic characteristics.

**Variable**	***N* (%) Mean ±SD**	**Drink unboiled tap water**	***p*-value**	**Willingness to use filter**	***p*-value**
		**Yes** ***n*** **(%)**		**Yes** ***n*** **(%)**	
**Gender**			0.118		0.231
Male	215 (43.00)	67 (31.16)		58 (26.98)	
Female	285 (57.00)	108 (37.89)		91 (31.93)	
**Age**	66.14 ± 9.19		**0.002**		**0.001**
45–60	112 (22.40)	51 (45.54)		49 (43.75)	
60–79	353 (70.60)	119 (33.71)		94 (26.63)	
≥80	35 (7.00)	5 (14.29)		6 (17.14)	
**Nationality**			0.275		0.258
Han	463 (92.60)	159 (34.34)		141 (30.45)	
Ethnic minority	37 (7.40)	16 (43.24)		8 (21.62)	
**Income (CNY)**			0.981		**0.037**
<1,000	34 (6.80)	13 (38.24)		5 (14.71)	
1,000–3,000	76 (15.20)	26 (34.21)		25 (32.89)	
3,000–5,000	152 (30.40)	55 (36.18)		38 (25.00)	
5,000–10,000	192 (38.40)	66 (34.38)		61 (31.77)	
>10,000	46 (9.20)	15 (32.16)		20 (43.48)	
**Education status**			**0.012**		**<0.001**
Primary school and below	360 (72.00)	138 (38.33)		90 (25.00)	
Middle school and above	140 (28.00)	37 (26.43)		59 (42.12)	

Chi-square test was used to compare the differences in all variables; P < 0.05, Significant results; SD, standard deviation. The bold values represent *P* < 0.05 (results are statistically significant).

### Water behavior and sanitary conditions of residents

More than half of residents (52.80%) get their tap water from spring water, 31.80% from stream water, 11.80% from lake water, and 3.60% from pond water. Nearly half of residents (45.60%) treated tap water at the household level before using it (such as sediment, filtration, disinfection, etc.). A small percentage of residents (6.80%) use bottled water, and more than half (59.60%) do not store water at home. Of the participants, 41.80% of the residents had the habit of drinking tea, while 58.20% did not. In terms of drinking water methods, 43.80% of the residents said they only drink water when they are thirsty, and more than half (56.20%) of the residents drink water at regular intervals. One in six respondents (15.60%) reported that they were suffering from or had kidney stones in the past year. Most residents (88.40%) are eager to gain knowledge about drinking water, and 11.60% had a negative attitude toward knowledge of drinking water. And nearly a quarter of residents (22.00%) use the village-type dry toilet. The chi-square test indicated that household water treatment, having kidney stones, and wanting to gain knowledge about drinking water were statistically significant with drinking unboiled tap water (*p* < 0.05), the tap water source, household water treat, water storage, and the toilet type were statistically significant with a willingness to use filters (*p* < 0.05) (Table 3).

**Table 3:** Drinking unboiled tap water and willingness to use filters by water behavior and sanitary conditions.

**Variable**	***N* (%)**	**Drink unboiled tap water**	***p*-value**	**Willingness to use filter**	***p*-value**
		**Yes** ***n*** **(%)**		**Yes** ***n*** **(%)**	
**The tap water source**			0.166		**0.001**
Lake water	59 (11.80)	25 (42.37)		23 (38.98)	
Pond water	18 (3.60)	3 (16.67)		12 (66.67)	
Stream water	159 (31.80)	60 (37.74)		48 (30.19)	
Spring water	264 (52.80)	87 (32.95)		66 (25.00)	
**Household water treat**			**0.016**		**<0.001**
Yes	228 (45.60)	67 (29.39)		102 (44.74)	
No	272 (54.40)	108 (39.71)		47 (17.28)	
**Drink bottled water**			0.682		0.059
Yes	34 (6.80)	13 (38.24)		15 (44.12)	
No	466 (93.20)	162 (34.76)		134 (28.76)	
**Water storage**			0.064		**0.001**
Yes	202 (40.40)	61 (30.20)		77 (38.12)	
No	298 (59.60)	114 (38.26)		72 (24.16)	
**Drink tea**					
Yes	209 (41.80)	64 (30.62)	0.082	67 (32.06)	0.350
No	291 (58.20)	111 (38.14)		82 (28.18)	
**The way of drinking water**			**<0.001**		0.068
Drink when thirsty	219 (43.80)	105 (47.95)		56 (25.57)	
Drinking water at regular interval	281 (56.20)	70 (24.91)		93 (33.10)	
**The toilet type**			0.428		**0.021**
Village-type dry toilet	110 (22.00)	35 (31.82)		23 (20.91)	
Hygienic toilet	390 (78.00)	140 (35.90)		126 (32.31)	
**Have kidney stones**			**<0.001**		0.948
Yes	78 (15.60)	43 (55.13)		23 (29.49)	
No	422 (84.40)	132 (31.28)		126 (29.86)	
**Want to gain knowledge about drinking water**			**<0.001**		0.055
Yes	442 (88.40)	131 (29.64)		138 (31.22)	
No	58 (11.60)	44 (75.86)		11 (18.97)	

Chi-square test was used to compare the differences in all variables. P < 0.05, Significant results. The bold values represent *P* < 0.05 (results are statistically significant).

### Respondents' perception of household tap water

In terms of residents' perception of water, the mean EC score for drinking unboiled tap water is 7.36 (SD = 0.30), and willing to use filters is 9.04 (SD = 0.40). The mean AS score for drinking unboiled tap water is 9.57 (SD = 0.09) and willing to use filters is 9.40 (SD = 0.10). Residents who drank unboiled tap water (6.10 ± 0.27) had lower PHR scores on average than those who did not drink unboiled tap water (8.04 ± 0.26). The average PHR score of residents willing to use filters (10.30 ± 0.43) was higher than that of residents unwilling to use filters (6.12 ± 0.17). Residents who drank unboiled tap water (13.28 ± 0.20) had higher OP scores on average than those who did not drink unboiled tap water (12.43 ± 0.17). The average OP score of residents willing to use filters (10.74 ± 0.28) was lower than that of residents unwilling to use filters (13.57 ± 0.13). Residents who drank unboiled tap water (12.63 ± 0.19) had higher WAT scores on average than those who did not drink unboiled tap water (12.63 ± 0.19). The average WAT score of residents willing to use filters (10.30 ± 0.28) was lower than that of residents unwilling to use filters (12.81 ± 0.13). Univariate analysis indicated that PHR, OP, and WAT were significantly associated with drinking unboiled tap water (*p* < 0.05). For willing to use filters, the difference between EC, AS, PHR, OP, and WAT were significant (*p* < 0.05) (Table 4).

**Table 4:** Drinking unboiled tap water and willingness to use filters by perception of household tap water.

**Variable**	**Drinking unboiled tap water**		***t***	***p***	**Willingness to use filters**		***t***	***p***
	**Yes (*n* = 175) Mean ±SD**	**No (*n* = 325) Mean ±SD**			**Yes (*n* = 149) Mean ±SD**	**No (*n* = 351) Mean ±SD**		
EC	7.36 ± 0.30	8.13 ± 0.24	1.905	0.057	9.04 ± 0.40	7.36 ± 0.21	−4.090	**<0.001**
AS	9.57 ± 0.09	9.57 ± 0.06	0.064	0.949	9.40 ± 0.10	9.64 ± 0.05	2.133	**0.033**
PHR	6.10 ± 0.27	8.04 ± 0.26	4.837	**<0.001**	10.30 ± 0.43	6.12 ± 0.17	−10.847	**<0.001**
OP	13.28 ± 0.20	12.43 ± 0.17	−3.027	**0.003**	10.74 ± 0.28	13.57 ± 0.13	10.650	**<0.001**
WAT	12.63 ± 0.19	12.63 ± 0.19	−3.200	**0.002**	10.30 ± 0.28	12.81 ± 0.13	9.434	**<0.001**

t-test was used to compare the differences; P < 0.05, Significant results.

SD, standard deviation; EC, Environment concern; AS, Area satisfaction; PHR, Perceived health risks; OP, Organoleptic Perceptions; WAT, Water authority trust.

The bold values represent *P* < 0.05 (results are statistically significant).

### Determinants of residents drinking unboiled tap water and willingness to using filters were based on binary logistic regression

Binary logistic regression analysis revealed that residents in the older-age group, 60–79 years old (OR: 0.510, 95% CI: 0.303–0.858), 80 and above years old (OR: 0.118, 95% CI: 0.038–0.365), drinking water at a regular interval (OR: 0.397, 95% CI: 0.257–0.612), want to gain knowledge about drinking water (OR: 0.198, 95% CI: 0.099–0.395), and whose PHR higher (OR: 0.847, 95% CI: 0.771–0.929) were less likely to drink unboiled tap water. However, residents having kidney stones (OR: 2.975, 95% CI: 1.708–5.253) were more likely to drink unboiled tap water (Table 5).

**Table 5:** Logistic regression model for rural residents of drinking unboiled tap water.

**Variable**	**OR**	**95% CI for OR**		***p***
		**Lower**	**Upper**	
**Age**
45–60	Ref.			
60–80	0.510	0.303	0.858	**0.011**
≥80	0.118	0.038	0.365	**<0.001**
**Education status**
Primary school and below	Ref.			
Middle school and above	0.641	0.387	1.059	0.082
**Household treat water**
No	Ref.			
Yes	0.695	0.447	1.079	0.105
**The way of drinking water**
Drink when thirsty	Ref.			
Drinking water at regular interval	0.397	0.257	0.612	**<0.001**
**Have kidney stones**
No	Ref.			
Yes	2.995	1.708	5.253	**<0.001**
**Want to gain knowledge about drinking water**
No	Ref.			
Yes	0.198	0.099	0.395	**<0.001**
**PHR**	0.847	0.771	0.929	**0.001**
**OP**	0.890	0.777	1.018	0.089
**WAT**	1.038	0.938	1.148	0.470

Ref., Reference. The bold values represent *P* < 0.05 (results are statistically significant).

In terms of willingness to use filters, logistic regression results showed that residents were elderly group, 60–79 (OR: 0.446, 95% CI: 0.244–0.815), 80 and above (OR: 0.228, 95% CI: 0.064–0.812), water storage (OR: 0.088, 95% CI: 0.026–0.300), and residents with a higher WAT (OR: 0.857, 95% CI: 0.769–0.956) were more likely to have a low willingness to use the filter. Residents who had a middle school and above (OR: 2.238, 95% CI: 1.289–3.883), household water treat (OR: 33.704, 95% CI: 9.726–116.791), and perceived more health risks (PHR) (OR: 1.106, 95% CI: 1.009–1.213) were more likely to be willing to use filters (Table 6).

**Table 6:** Logistic regression model for rural residents of willingness to use filter.

**Variable**	**OR**	**95% CI for OR**		***p***
		**Lower**	**Upper**	
**Age**
45–60	Ref.			
60–80	0.446	0.244	0.815	**0.009**
≥80	0.228	0.064	0.812	**0.022**
**Income (CNY)**
<1,000	Ref.			
1,000–3,000	2.542	0.663	9.743	0.174
3,000–5,000	1.680	0.466	6.063	0.428
5,000–10,000	2.086	0.593	7.340	0.252
>10,000	4.026	0.951	17.048	0.059
**Education status**
Primary school and below	Ref.			
Middle school and above	2.238	1.289	3.883	**0.004**
**household water treatment**
No	Ref.			
Yes	33.704	9.726	116.791	**<0.001**
**The tap water source**
Lake water	Ref.			
Pond water	0.576	0.116	2.868	0.501
Stream water	0.506	0.215	1.189	0.118
Spring water	0.688	0.314	1.510	0.351
**Water storage**
Yes	Ref.			
No	0.088	0.026	0.300	**<0.001**
**The toilet type**
Village-type dry toilet	Ref.			
Hygienic toilet	1.759	0.897	3.447	0.100
**EC**	1.007	0.946	1.073	0.822
**AS**	1.024	0.810	1.294	0.845
**PHR**	1.106	1.009	1.213	**0.031**
**OP**	0.867	0.750	1.003	0.055
**WAT**	0.857	0.769	0.956	**0.006**

Ref., Reference. The bold values represent *P* < 0.05 (results are statistically significant).

## Discussion

Drinking water behavior reflects residents' awareness and philosophy of health and drinking water sanitation. However, few studies have examined the factors that influence drinking water behavior in China, especially in rural areas where drinking water safety is a major concern. We conducted a cross-sectional survey in six villages in Tengchong County, Yunnan Province, where water quality is poor and SCWS is common in China, to understand the drinking of unboiled tap water by middle-aged and elderly rural residents and the possibility of promoting filters. The results showed that a large number of middle-aged and elderly residents also drank unboiled tap water to some extent (35%). In addition, only 7.6% of the residents in our survey have any drinking water filtration facilities in their homes, and only 29.8% of them are willing to use filters. Many factors affect residents to drink unboiled tap water and the willingness to use filters, so explore the reasons and formulate measures and strategies to eliminate the behavior of middle-aged and elderly rural residents to drink unboiled tap water. At the same time, understanding the willingness of residents to treat water with filters is of great significance for the construction of rural drinking water safety.

Our study found that the elderly were more likely not to drink unboiled tap water, and the older they were, the less likely they were to drink unboiled tap water. This may be because compared with the middle-aged, the elderly generally have lower body resistance, are more likely to be affected by various external risk factors, and suffer from a high proportion of chronic diseases. Years of treatment and medication experience will also make them pay more attention to health (27, 28). Generally speaking, the level of education often reflects the level of health knowledge (29), but in our regression model, the relationship between education level and drinking unboiled tap water is not significant (*p* = 0.082). This may be due to the fact that middle-aged and elderly rural residents generally have a low level of education. In the appropriate stage of education, they are trapped in the backward social and economic development in China and lack educational resources (30). Most of them do not receive systematic elementary education, resulting in generally low health literacy (31, 32). However, it should be noted that residents with higher education levels (middle school and above) still drank less unboiled tap water than those with lower education levels (primary school and below) (26.43% vs. 38.33%). In addition, residents who want to gain knowledge about drinking water are more likely not to drink unboiled tap water. This is because human behavior is heavily influenced by personal and situational motivational factors, which is the core idea of self-determination theory (33, 34). When residents want to acquire knowledge about drinking water, there is an intrinsic motivation to engage in healthy behaviors, which makes sense why residents who actively acquire knowledge about drinking water are more likely to avoid drinking unboiled tap water. Meanwhile, respondents who intake water at the regular interval were more likely to avoid unboiled tap water than those who drank only when they were thirsty. The scientific way of drinking water is at regular intervals intaking water, especially the elderly should take sufficient water every day, and those who drink only when they are thirsty often neglect the health of drinking water (35, 36), so they had more possibility of drinking unboiled tap water. We also found that residents with kidney stones were more likely to drink unboiled tap water. At present, the effect of drinking water quality on kidney stone disease has been a long-term debate (37). It is generally believed that kidney stones are related to the amount of water consumed, and the simplest and most important lifestyle change to prevent stone disease is to drink more water/fluid (38). While people who drink at regular intervals tend to intake enough water throughout the day, people who drink only when they are thirsty tend not to intake enough water. And studies have found that residents who drink hard groundwater water have a higher risk of kidney stones than those who drink treated water (39), and boiling can effectively reduce the hardness of drinking water (40). No wonder residents with kidney stones are more likely to drink unboiled tap water. We also found that residents with higher perceived health risk (PHR) were less likely to drink unboiled tap water. Previous studies have found that residents avoid unsafe water at home by boiling or filtering or purchasing bottled water based on their perception of water quality (41). Therefore, the health risk perception of drinking water can reflect residents' drinking water behavior to a certain extent.

Our study found that differences in age, education, and household income in influencing water filter use choices are both similarities and differences with western countries (18, 25, 42). These factors show the characteristics of Chinese tradition and social and economic background. Older people were more likely than middle-aged people to be reluctant to use filters because elderly people in rural China have low incomes and generally low levels of education (31), as well as traditional lifestyles and long-term use of boiled tap water. In previous studies, higher education levels were also more likely to choose alternative water (filters and bottled water) (19, 42). And our research also confirms this, with more educated residents more likely to be willing to use filters. Although family income was not significant in our regression, in univariate analysis, the proportion of residents with higher household income willing to use filters was still higher (*P* < 0.037). In addition, in self-perception theory, it is generally believed that a person's behavior can express his or her true attitude (43). Therefore, residents' behavior of treating water at home reflects their attitude of wanting to improve drinking water sanitation, which explains that in our survey, residents with household water treatment behavior are more likely to be willing to use filters. Many residents in China's rural areas have the habit of water storage. However, this behavior has certain health risks, and it is easy to cause secondary pollution (44). Among all the residents who can store water, 51.49% use plastic buckets with no covers to store water, while the rest use self-built water cellars and tanks, where the safety of water is not guaranteed. It can be seen that these residents who have the habit of water storage often lack the awareness of healthy water use, so they are more likely not willing to use filters to improve water sanitation. PHR was also an important factor in our second regression. Similar to the Kenyan study, as the perceived risk to water increases, households are more likely to dispose of unimproved drinking water (45), and thus more likely to be willing to use filters. Residents' satisfaction with water supply institutions often affects residents' views on domestic water supply, both in water quality perception and health risk perception, as well as residents' choice of water and willingness to treat water at the household level (25, 46). Our results showed that the higher the WAT, the lower the willingness of residents to use filters.

This is the first survey of water use behavior and willingness of middle-aged and elderly residents in rural China. In recent years, China has made great achievements in strengthening infrastructure construction and promoting projects to upgrade drinking water and toilets in rural areas. However, there are still considerable problems with drinking water for rural residents, especially the middle-aged and elderly with low education, low income, and ill-informed. A large proportion of them drink unboiled tap water. At the same time, residents showed a lower willingness to use filters. This is mainly related to the drinking water status, the health literacy of residents and the stage of social and economic development in rural China. In addition, understanding residents' perception of drinking water can provide a more comprehensive understanding of residents' drinking water behavior. Therefore, policymakers should pay more attention to the improvement of health knowledge and cultural literacy of middle-aged and elderly rural residents (47), especially the middle-aged group should not be neglected. Health education can be expanded to indirectly affect the drinking water behavior of middle-aged and elderly people by guiding families and children (48). At the same time, regular health lectures were carried out to guide and standardize the drinking water behavior of middle-aged and elderly residents, and to eliminate the behavior of drinking unboiled tap water. In addition, promote safe, effective and economic filtration and water purification facilities in rural SCWS areas to improve access to safe drinking water. Most importantly, water authorities and the CDC should be urged to strengthen the management and supervision of drinking water quality to protect public health.

It is important to mention some limitations of our study. Firstly, our analysis used a cross-sectional study design that only indicates the current domestic water behavior, and the results cannot infer causality. Secondly, limitation of sample selection, research results only represent the behavior and will of residents in this survey area, not other regions. Additionally, we only focus on middle-aged and elderly living in rural areas, but there are still large left-behind children who also face the same problem. Moreover, participants self-reported that the relevant information may be biased, and we did not explore the influence of chemical indicators of water quality on the results. These limitations also point out the direction for our further research.

## Conclusion

We found that a certain proportion of permanent middle-aged and elderly residents in rural areas still drink unboiled tap water, especially those in middle age, and there is a low willingness to use filters. Economic and cultural level, health literacy, drinking water behavior, and drinking water perceived are the important factors influencing the willingness of middle-aged and elderly residents to drink unboiled tap water and use filters. Residents over 60 years of age who have regular drinking behavior, want knowledge about drinking water, and have a high level of PHR are more likely not to drink unboiled tap water. Residents with a middle school education or above, household water treatment behavior, and a high level of PHR are more likely to be willing to use filters. Therefore, the government and CDC should strengthen the popularization of drinking water knowledge for middle-aged and elderly rural residents, regularly carry out health education and other related measures, strengthen the regulation of middle-aged and elderly residents' drinking water behavior, eliminate the behavior of drinking unboiled tap water. Promote safe, economical and effective domestic water filtration facilities in rural SCWS areas to ensure public health safety.

## Data availability statement

The raw data supporting the conclusions of this article will be made available by the authors, without undue reservation.

## Ethics statement

The studies involving human participants were reviewed and approved by Ethical Committee of Dalian Medical University. The patients/participants provided their written informed consent to participate in this study.

## Author contributions

YD and LZ: conceptualization. YD and YP: data curation. YD: formal analysis and writing—original draft. RW, XC, JX, and MS: investigation. YD, HJ, and LZ: methodology. LZ: project administration, resources, and writing—review and editing. RW and HJ: software. XC, JX, and YC: supervision. YC and YP: validation. MS: visualization. All authors contributed to the article and approved the submitted version.

## Conflict of interest

The authors declare that the research was conducted in the absence of any commercial or financial relationships that could be construed as a potential conflict of interest.

## Publisher's note

All claims expressed in this article are solely those of the authors and do not necessarily represent those of their affiliated organizations, or those of the publisher, the editors and the reviewers. Any product that may be evaluated in this article, or claim that may be made by its manufacturer, is not guaranteed or endorsed by the publisher.

## Abbreviations

SCWS: small centralized water supply
EC: Environment concern
HWT: household water treatment
AS: Area satisfaction
PHR: Perceived health risks
OP: Organoleptic Perceptions
WAT: Water authority trust
SD: standard deviations
VIF: variance inflation factor
OR: Odds ratio
CIs: confidence intervals
MHI: monthly household income
Ref.: Reference
CDC: Centers for Disease Control.
